# Single-cell RNA-seq data normalization: A benchmarking study

**DOI:** 10.1371/journal.pone.0335102

**Published:** 2025-12-18

**Authors:** Qinyu Ge, Yuqi Sheng, Junru Lu, Yuwei Yang, Min Pan

**Affiliations:** 1 State Key Laboratory of Digital Medical Engineering, School of Biological Science and Medical Engineering, Southeast University, Nanjing, China; 2 School of Medicine, Southeast University, Nanjing, China; Beijing Chao-Yang Hospital Capital Medical University: Beijing Chaoyang Hospital, CHINA

## Abstract

This study examines the noise and biases introduced by technical factors in single-cell RNA sequencing (scRNA-seq) data, presenting a thorough benchmarking analysis of six widely utilized normalization methods. The evaluation of these methods is conducted from three perspectives: cell clustering, differential expression analysis, and computational resource requirements, utilizing seven real datasets alongside four simulated datasets. The findings indicate that Dino excels in clustering 10 × datasets and those with a substantial number of cells, while scTransform demonstrates strong performance with datasets produced through full-length library preparation protocols. Additionally, SCnorm is identified as suitable for small-scale datasets. This research serves as a significant reference for scholars in selecting appropriate normalization tools, thereby enhancing the accuracy and reliability of subsequent analyses of scRNA-seq data.

## Introduction

In recent years, single-cell RNA sequencing (scRNA-seq) has emerged as a pioneering technology within the life sciences, providing researchers with unparalleled insights into cellular heterogeneity and the identification of rare cell populations [1,2]. Luo et al. integrated scRNA-seq data from peripheral blood cells of five different autoimmune diseases and revealed 18 different subsets of immune cells [3]. By enabling high-resolution transcriptomic analysis at the single-cell level, scRNA-seq has significantly enhanced our understanding of various biological processes, including cellular development and disease progression [4–6]. Researchers have revealed disease development and identified novel therapeutic targets in complex diseases such as tumors [6,7], cerebrovascular diseases [8] and Alzheimer’s disease [9] by scRNA-seq. However, the rapid evolution of this technology has also presented substantial challenges in data analysis, primarily due to the intricate and distinctive nature of scRNA-seq datasets [10,11].

The technical attributes of scRNA-seq inherently complicate the accurate interpretation of data. The limited RNA content in individual cells necessitates amplification, a process that introduces considerable bias owing to variable efficiency across different genes and cells, this process introduces technical noise [12–14]. Additionally, cell cycle effects can lead to significant variability in gene expression, resulting in systematic biases that may not correlate with biological phenotypes [15]. Discrepancies in library size (sequencing depth) among cells, which can arise from stochastic or systematic errors during library preparation, and at the same time trigger the dropout events. This is also the main reason for the high sparsity of scRNA-seq data, further hinder cross-sample comparisons of gene expression levels. The generation of noise and the distortion of biological signals caused by these technical artifacts obscure meaningful insights and impede subsequent analyses [16–21].

To address these challenges, data normalization has become an essential preprocessing step in scRNA-seq analysis, prompting extensive research into the development of robust normalization methods [22–24]. Despite the introduction of numerous approaches aimed at mitigating technical biases and enhancing data quality, a universally applicable method has yet to be established, and its effectiveness remains unverified across diverse experimental contexts. The selection of an appropriate normalization tool is complicated by inherent differences in algorithmic principles, performance metrics, and applicability [25]. Some methods may overcorrect, inadvertently removing genuine biological signals, while others may under correct, failing to adequately address technical variability, both of which compromise the reliability of downstream analyses [26–28]. Moreover, the expanding range of applications for scRNA-seq necessitates the development of normalization methods that are adaptable, accurate, and computationally efficient.

Given the heterogeneity of available normalization techniques and the lack of a systematic benchmarking process, there is an urgent need for comprehensive evaluations to guide the selection of appropriate methods. The present study systematically compares six widely utilized scRNA-seq normalization methods across seven real-world datasets and four simulated datasets [27–31], employing multiple evaluation metrics, including cell clustering accuracy, differential expression analysis, and computational resource consumption. The objective of this study is to provide researchers with recommendations for selecting optimal normalization strategies for scRNA-seq, thereby maximizing the potential of this technology to reveal novel biological insights and advance the field of single-cell transcriptomics.

## Materials and methods

### Datasets

#### Real datasets.

The current investigation has compiled a total of seven authentic single-cell transcriptome datasets, which are considered crucial for ensuring the rigor and validity of the findings. The datasets include six derived from various species and one pertaining to a pathological condition (Table 1).

**Table 1:** Seven real datasets for benchmarking the standardized method of scRNA-seq.

Dataset_ID	Name	Protocol	Tissue	Features	Samples	Cell types	Link
**Dataset1**	Zeisel	STRT-Seq UMI	Brain	19972	3005	9	GSE60361
**Dataset2**	Grubman	Smart-Seq2	Brain	10850	13214	8	GSE138852
**Dataset3**	Nestorowa	Smart-Seq2	Blood	36421	1920	8	GSE81682
**Dataset4**	Tabula_Trachea	10× Genomics UMI	Trachea	23433	11269	7	10.6084/m9.figshare.5715025
**Dataset5**	Tabula_Marrow	10× Genomics UMI	Marrow	23433	3652	8	10.6084/m9.figshare.5715025
**Dataset6**	Tabula_Lung	10× Genomics UMI	Lung	17277	5449	10	10.6084/m9.figshare.5715025
**Dataset7**	Alma	10× Genomics	Brain	27998	8449	56	mousebrain.org

Dataset 1 (Zeisel) consists of 3,005 brain cells sourced from adult mice through microdissection, utilizing the STRT-Seq UMI library preparation protocol. This method incorporates unique molecular identifiers to mitigate PCR amplification biases. The raw data is accessible via the GSE60361 project on NCBI and encompasses nine distinct cell types.

Dataset 2 (Grubman) includes 13,214 cells isolated from the entorhinal cortex of individuals diagnosed with Alzheimer’s disease, alongside age-matched controls, achieved through fluorescence-activated cell sorting. The application of SMART-seq2 library preparation technology allows for full-length transcript coverage, thereby enhancing the detection of gene expression. This dataset can be found in the GSE138852 project on NCBI and also contains nine cell types.

Datasets 3 (Nestorowa) focuses on the isolation of hematopoietic stem and progenitor cells from mouse bone marrow. After a thorough screening process based on criteria such as cell viability and gene detection quality, 1,920 cells and 36,421 genes were selected for further analysis. The raw data is stored on a designated platform and includes eight primary cell types.

Datasets 4–6 (Tabula Muris) comprise sub-datasets from the mouse single-cell atlas project, collected from three distinct anatomical sites: the trachea, bone marrow, and lung. All subjects employed the 10× Genomics UMI library preparation method, which has been shown to facilitate high-throughput single-cell capture through microfluidic technology (Smith et al., 2022). The data, retrieved from figshare, includes 11,269, 3,652, and 5,449 cells, respectively, each with well-annotated cell types.

Finally, Dataset 7 (Alma) features 8,449 hippocampal cells from mice, prepared using the 10× Genomics library preparation protocol. Following processing, this dataset comprises 56 sub-cell types and is available for download from mousebrain.org.

#### Simulated datasets.

Four simulated datasets were generated utilizing the Splatter software package [32], with an initial gene count of 20,000 and nine major cell types. The dropout rates were manipulated at two levels, 0.25 and 0.5, to simulate varying degrees of data loss, reflecting the information loss that can occur due to factors such as incomplete transcript capture or amplification failures in empirical studies. Additionally, two cell size conditions, comprising 5,000 and 20,000 cells, were established to assess the robustness of analytical methods across different sample sizes. The primary objective of these simulated datasets was to systematically evaluate the effects of normalization techniques on subsequent clustering and differential expression analyses. The known true number of differentially expressed genes within the simulated datasets further enabled an assessment of the efficacy of each analytical method in accurately identifying these genes.

### Single-cell transcriptome data normalization methods

Quality control of the raw data was conducted using the Seurat software package (version 4.3.0) [33]. Cells exhibiting fewer than 200 expressed genes, a total count of fewer than 500, or a mitochondrial gene ratio exceeding 10% were excluded from the analysis. The data subsequently underwent preprocessing normalization via logarithmic transformation.

Data normalization plays a crucial role as a preprocessing step in scRNA-seq analysis because it addresses technical variability. This investigation compared six widely utilized normalization methods. The log method, which serves as the default approach in Seurat, compresses the data’s dynamic range by transforming it into the form log₁₀(x + 1) (where x represents the original gene expression count), thereby approximating a normal distribution of the data. LIGER (version 1.2.3) [31] employs integrated non-negative matrix factorization to align disparate datasets within a shared low-dimensional space, effectively mitigating batch effects and technical discrepancies. Dino (version 1.1.0) [28] utilizes a negative binomial mixture model to characterize the mean-variance relationship of gene expression, thereby addressing technical noise and intercellular variability. SCnorm (version 1.18.0) [30] applies quantile regression to normalize gene expression by fitting the quantiles of reference cells, while scTransform (version 0.4.4) [29] employs regularized negative binomial regression and variance-stabilizing transformation to account for factors such as sequencing depth. Lastly, scran (version 1.34.0) [34] implements a deconvolution strategy to correct for cell-specific biases by estimating the size factor for each individual cell.

Post-normalization, the FindVariableFeatures function within the Seurat software package was utilized to identify 2,000 highly variable genes based on the coefficient of variation and the mean-variance relationship. Dimensionality reduction was achieved through principal component analysis (PCA), retaining the first 50 principal components as determined by the scree plot of eigenvalues. The Louvain algorithm was subsequently applied for cell clustering, with the resolution parameter set between 0.5 and 1.0. Visualization was performed using the uniform manifold approximation and projection (uMAP) algorithm. For downstream differential expression (DE) analysis, marker genes for each cluster were identified using the Wilcoxon rank-sum test. DE analysis was conducted using cluster-assigned labels rather than ground-truth labels. To account for multiple testing, p-values were adjusted using the Benjamini-Hochberg procedure, with a significance threshold of p < 0.05. These settings ensure reproducibility and provide a standardized evaluation framework for comparing normalization methods.

### Evaluation system for single-cell transcriptome data normalization methods

The efficacy of the normalization methods was assessed from three distinct perspectives: cell clustering, differential expression analysis, and computational resource requirements. The evaluation of cell clustering performance utilized a variety of metrics, including the Adjusted Rand Index (ARI) [35], Adjusted Mutual Information (AMI) [36], V-Measure, Fowlkes-Mallows Index (FMI) [37], and Silhouette Score (sc) [38]. These indices, with the exception of the Silhouette Score which ranges from −1 to –1, span from 0 to 1, where higher values signify enhanced clustering performance. In terms of differential expression analysis, performance was gauged using precision, recall, and F1-score as key metrics. Precision reflects the accuracy of the analysis results, recall indicates the sensitivity of the analytical method, and the F1-score provides a holistic assessment of performance. All metrics in this context also range from 0 to 1, with elevated values denoting superior analytical performance. The evaluation of computational resource requirements involved monitoring peak memory usage and CPU execution time. The experiments were conducted on a server equipped with dual AMD EPYC 7742 processors (64 cores, 2.25 GHz), 512 GB of RAM, and operating on Ubuntu 18.04.6 LTS. Resource consumption measurements were facilitated by system-integrated tools.

## Results

### Benchmarking results of scRNA-seq normalization methods on real datasets

**Fig 1:**
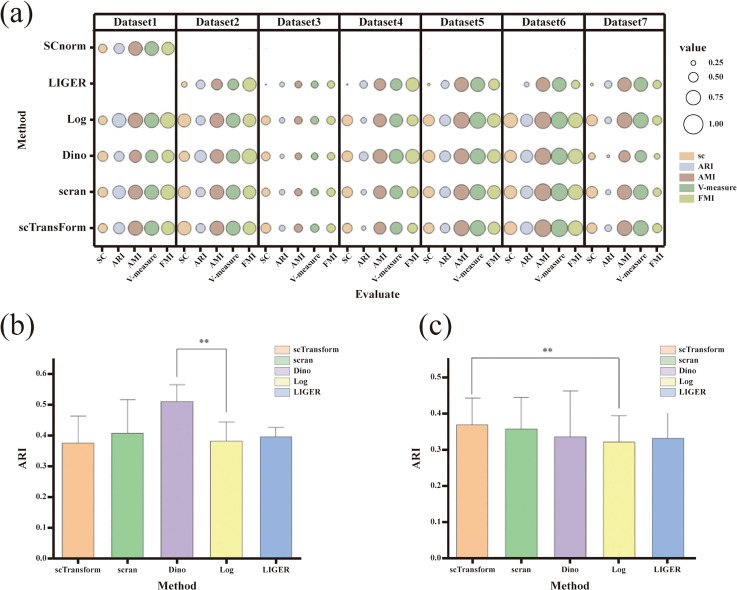
Illustrates the benchmark test results of the single-cell RNA sequencing (scRNA-seq) standardization method applied to a real dataset. (a) provides a comprehensive overview of these benchmark results, where the size of each bubble corresponds to the value of the evaluation metric associated with the vertical axis for the respective dataset, while the varying colors denote different evaluation metrics. (b) presents the Adjusted Rand Index (ARI) values achieved by each scRNA-seq standardization method across datasets 4, 5, and 2. (c) displays the ARI values obtained from the application of each scRNA-seq standardization method on datasets 2 and 3.

The efficacy of six normalization methods for cell clustering was assessed using seven real datasets. As illustrated in Fig 1a, the Dino normalization method demonstrated exceptional performance in 10× datasets, particularly in Dataset4 and Dataset5, as well as in datasets characterized by a high cell count, such as Dataset2. The ARI obtained in this study was significantly greater than that of the conventional Log Normalization method (p < 0.05), as shown in Fig 1b. Furthermore, the scTransform normalization method exhibited comparable or superior clustering performance relative to the Log method in datasets derived from the SMART-Seq2 full-length library preparation protocol (e.g., Dataset2 and Dataset3), resulting in a significantly higher ARI index (p < 0.05), as depicted in Fig 1c.

**Fig 2:**
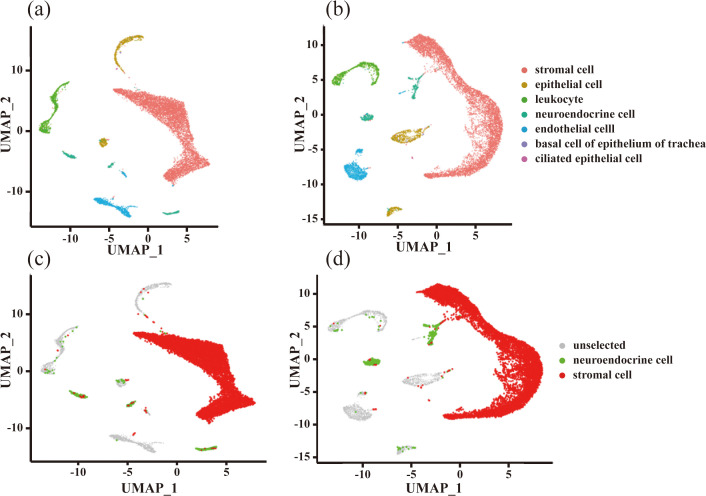
Presents the results of UMAP dimension reduction utilizing both the Dino and Log standardization methods. (a) illustrates the annotation outcomes for each cell type in the UMAP representation employing the Dino standardization method within dataset 4. (b) displays the annotation results for each cell type in the UMAP visualization using the Log standardization method in the same dataset. (c) focuses on the labeling of neuroendocrine and stromal cells in the UMAP representation, again utilizing the Dino standardization method in dataset 4. Finally, (d) depicts the labeling of neuroendocrine and stromal cells in the UMAP visualization, this time employing the Log standardization method within dataset 4.

**Fig 3:**
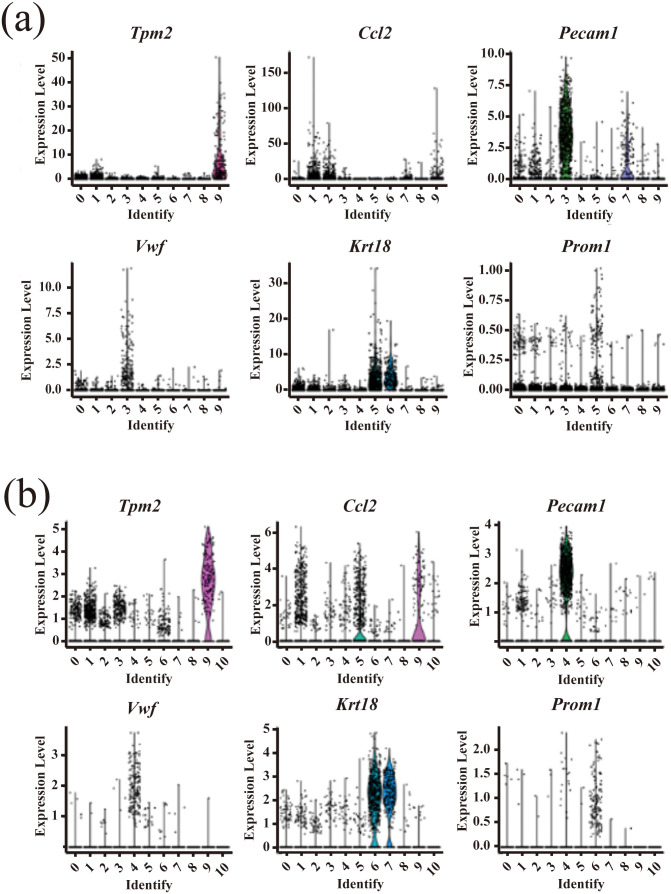
Illustrates the expression of marker genes within Dataset 4. **(a)** Violin plots depicting the expression levels of each marker gene across various clusters in Dataset 4, utilizing the Dino standardization method; **(b)** A violin plot representing the expression levels of each marker gene across clusters in Dataset 4, employing the Log standardization method.

As demonstrated in Dataset 4, the Dino normalization method produced more favorable outcomes in cell clustering compared to the Log method, as illustrated in Fig 2. The UMAP plot indicated a reduced likelihood of misclassifying neuroendocrine cells and stromal cells within the same cluster. Specifically, the number of misclassified cells decreased from 40 and 46 with the Log method to 28 and 31 with the Dino method (refer to Fig 2c and 2d). Additionally, the Dino normalization method enhanced the expression levels of marker genes such as *Tpm2, Ccl2, Pecam1, Vwf, Krt18*, and *Prom1* which associated with neuroendocrine cells, endothelial cells, epithelial cells, among others (see Fig 3). Compared with conventional Log normalization, Dino produced a more coherent and biologically interpretable clustering structure by preventing the artificial subdivision of homogeneous populations—such as stromal and neuroendocrine cells—often caused by residual variance related to library size effects. By modeling gene expression conditional on sequencing depth, Dino effectively reduced technical noise while preserving genuine biological variability, resulting in sharper and more distinct expression patterns of cell-type–specific marker genes. This improvement reflects a more accurate recovery of true biological heterogeneity rather than an artificial inflation of expression levels introduced by normalization bias.

**Fig 4:**
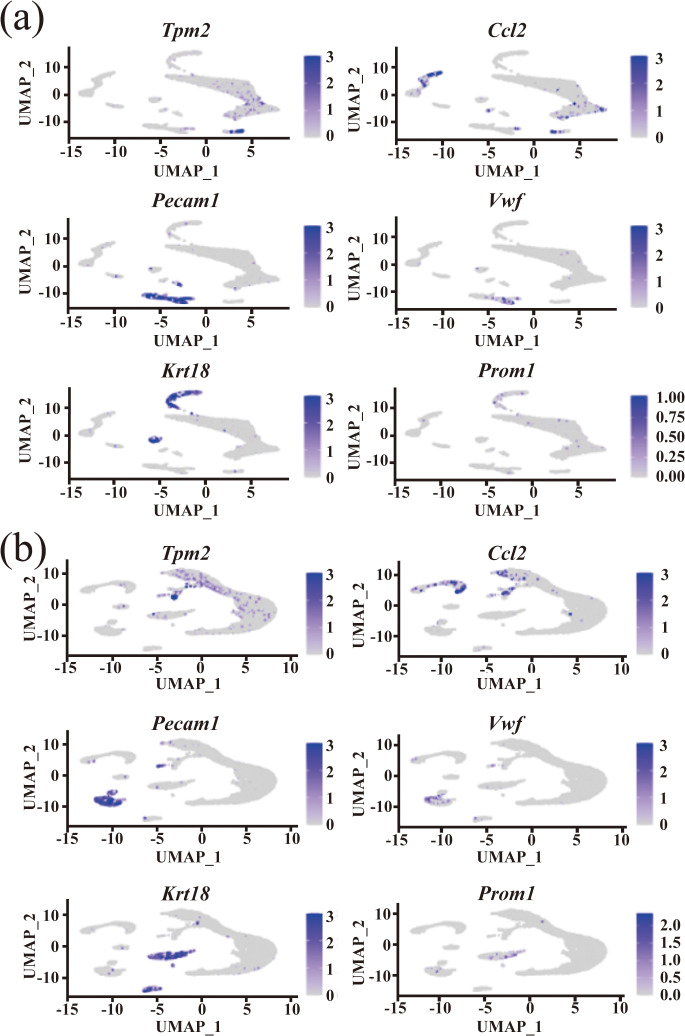
Presents the distribution of marker gene expression in Dataset 4. **(a)** The distribution of expression levels for each marker gene within the UMAP representation of Dataset 4, based on the Dino standardization method; **(b)** The distribution of expression levels for each marker gene within the UMAP representation of Dataset 4, utilizing the Log standardization method.

Moreover, the distribution of these expression levels closely mirrored the actual distributions of the respective cell types (refer to Fig 4). Specifically, in Fig 4, the violin plots illustrate that marker genes such as Tpm2, Ccl2, and Vwf show sharper, more distinct peaks under Dino normalization, with lower cross-group expression overlap compared to Log normalization. This indicates that Dino better preserves cell-type–specific expression boundaries, leading to higher intra-cluster homogeneity and reduced inter-cluster leakage of signal. In contrast, Log normalization tends to produce flatter and more dispersed distributions, suggesting weaker contrast between marker-positive and marker-negative cell types. Although we did not quantify this difference numerically, the clearer separation of marker expression patterns in Dino-normalized data visually supports its superior ability to reflect true biological distinctions among cell types.

### Benchmarking results of scRNA-seq normalization methods on simulated datasets

**Fig 5:**
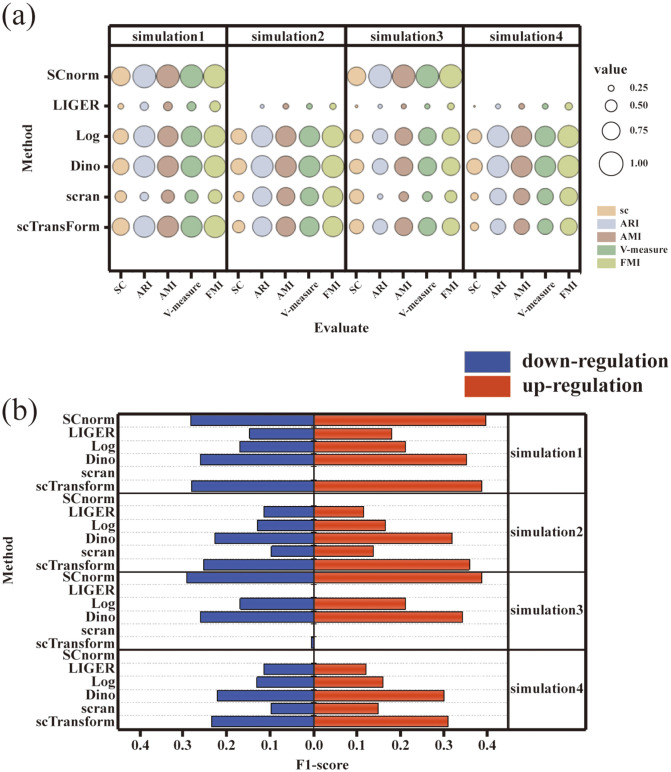
Illustrates the benchmark test outcomes of the single-cell RNA sequencing (scRNA-seq) standardization methods across various simulated datasets. (a) displays the results of cluster evaluation for each standardization method, where the size of the bubbles corresponds to the value of the evaluation index associated with the vertical coordinate for each dataset, and distinct colors denote different evaluation indices. (b) presents the benchmark test results for differential expression analysis conducted by each standardization method, specifically calculating the F1 scores for both up-regulated and down-regulated differentially expressed genes in comparison to the true differentially expressed genes.

As illustrated in Fig 5a, the Dino normalization method exhibited superior performance in cell clustering assessments across four simulated datasets, as indicated by its significantly higher clustering evaluation metrics when compared to the Log normalization method. The scTransform method showed optimal performance in simulated datasets characterized by low dropout rates, specifically Simulated Datasets 1 and 2. In contrast, its effectiveness was notably reduced in datasets with high dropout rates. The SCnorm method proved effective for small-scale datasets, such as Simulated Datasets 1 and 3, but was unable to successfully complete the normalization process for larger datasets.

In the evaluation of differential expression analysis performance, the results were consistent with those observed in cell clustering performance (see Fig 5b). The Dino method achieved higher F1-scores for differentially expressed genes across all four simulated datasets. The scTransform method maintained robust performance in datasets with low dropout rates; however, its effectiveness was significantly compromised in datasets characterized by high dropout rates and a limited number of cells. The SCnorm method demonstrated efficacy in small-scale datasets.

It should be noted that the overall F1-scores were relatively low, with a maximum around 0.4, mainly due to the conservative evaluation settings used in this study. Specifically, differential expression analysis was conducted using the Wilcoxon rank-sum test implemented in Seurat, with ground-truth cell-type labels provided by the Splatter simulation. A stringent false discovery rate (FDR < 0.05) and fold-change cutoff (|log₂FC| > 1) were applied to ensure high-confidence gene detection, which inherently reduced recall and thus lowered the F1-scores. Moreover, the simulated datasets were designed with moderate effect sizes and realistic dropout noise, making DE detection more challenging. Therefore, the relatively low F1-scores reflect strict evaluation criteria under realistic single-cell conditions rather than deficiencies in the normalization methods themselves.

### Evaluation of computational resource requirements of scRNA-seq normalization methods

**Fig 6:**
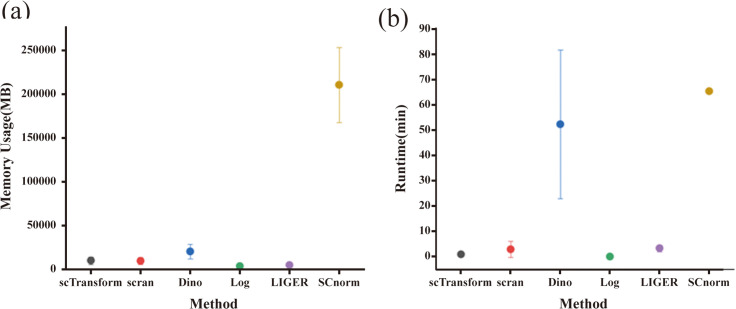
Illustrates the computational resource utilization associated with various single-cell RNA sequencing (scRNA-seq) data standardization techniques across all datasets. (a) presents a graph depicting the peak memory usage intervals for each data analysis method across the entire set of datasets. (b) displays a graph representing the runtime intervals for each data analysis method across all datasets.

As illustrated in Fig 6, the results of the evaluation concerning the computational resource requirements are presented. Among the various datasets subjected to benchmarking, the SCnorm method exhibited the highest memory demand, with an average peak memory usage surpassing 200 GB. The Dino method followed as the second most memory-intensive approach, while the remaining single-cell transcriptome data normalization methods required less than 32 GB of peak memory. Notably, there was no significant increase in memory requirements as the dataset size grew, making these methods suitable for use on standard home computers. In terms of computational time, the SCnorm method was the most time-consuming and frequently encountered performance issues, often failing to complete tasks on several large-scale datasets. In contrast, most normalization methods, excluding SCnorm, supported parallel computing and successfully processed all datasets, indicating a high level of compatibility. The Dino normalization method displayed adequate computational performance; however, its computation time significantly increased with larger dataset sizes.

## Discussion

The present study conducted a systematic evaluation of the performance of six widely employed normalization techniques across a variety of single-cell RNA sequencing datasets, encompassing both real-world and simulated data. The results indicate subtle variations in the effectiveness of these methods, which are influenced by the distinct characteristics of the datasets and the specific goals of the analysis.

### Clustering performance

The findings suggest that Dino demonstrates superior clustering capabilities for 10 × datasets and those characterized by a high cell count. This notable performance is attributed to the method’s adept representation of the mean-variance relationship in gene expression through a negative binomial mixture model, which effectively mitigates technical noise and intercellular variability. As a result, Dino facilitates an accurate representation of cellular heterogeneity, as evidenced by enhanced clustering metrics, particularly in datasets such as Tabula Muris (Datasets 4 and 5) and Grubman (Dataset 2).

Conversely, scTransform exhibits strong performance with datasets generated using the SMART-Seq2 full-length library preparation protocol. The integration of regularized negative binomial regression and variance-stabilizing transformation enables scTransform to effectively adjust for sequencing depth and cell-specific biases, thereby preserving more biological signals and improving clustering accuracy, as demonstrated in datasets like Zeisel (Dataset 1) and Grubman (Dataset 2).

SCnorm is identified as a viable option for smaller datasets, as indicated by its robust performance in simulated datasets with a limited number of cells. However, the high computational complexity and memory demands associated with the quantile regression strategy employed by SCnorm present challenges for its application to larger datasets.

### Differential expression analysis

The results of the differential expression analysis reflect the trends observed in clustering performance. Dino consistently achieves high F1-scores for differentially expressed genes across all simulated datasets, underscoring its strong capacity to identify biologically significant changes in gene expression.

scTransform maintains robust performance in datasets characterized by low dropout rates, as evidenced by its high F1-scores in simulated datasets 1 and 2. However, its effectiveness diminishes in datasets with considerable data loss, revealing a limitation in its ability to manage high levels of technical noise.

SCnorm demonstrates effectiveness in small-scale datasets, as indicated by its high F1-scores in simulated datasets 1 and 3. Nevertheless, its performance is constrained by the high computational complexity and memory requirements associated with its quantile regression approach.

### Computational resource requirements

The computational resource requirements of the normalization methods exhibit considerable variability. SCnorm, with its substantial memory consumption and prolonged computation time, is less suited for large-scale datasets due to the computational complexity of its quantile regression strategy. In contrast, alternative methods such as Dino, scTransform, and scran display more moderate resource requirements and support parallel computing, rendering them more appropriate for large-scale data analysis.

### Future development

In this study, we evaluated six commonly used standardization methods. Most of these are reference-free approaches based on algorithms that analyze the intrinsic data structure. These methods could be combined with reference-based standardization techniques in future studies to enable a more comprehensive evaluation of single-cell data. For example, Liu and his collaborators recently developed a standardized method utilizing mitochondrial mRNA as an internal reference [39], which has demonstrated significant advantages over conventional exogenous references. This approach offers a promising direction for our research. Furthermore, integrating a composite internal reference with data-structure algorithms and standardizing cross-modal multi-omics datasets are key areas for future development.

In addition, as scRNA-seq datasets continue to expand to millions of cells, the computational scalability and memory efficiency of normalization methods will become increasingly important. While our current benchmarking was performed on datasets of moderate size (ranging from thousands to tens of thousands of cells), future work will systematically evaluate how these normalization algorithms scale with increasing data volume. In particular, methods such as Dino and scTransform, which employ model-based or regularized frameworks, may be more computationally efficient and stable in large-scale settings. A comprehensive assessment of their scalability and performance trade-offs will be an important next step.

## Conclusions

The current investigation conducted a systematic assessment of the performance of six widely utilized normalization techniques by benchmarking them against various single-cell transcriptome datasets. The results revealed that the effectiveness of these normalization methods is contingent upon the specific characteristics of the datasets in question. Notably, the Dino Normalization method demonstrated superior performance in clustering analyses of 10× datasets and those with a large number of cells. Additionally, it exhibited strong capabilities in differential expression analysis on simulated datasets. In contrast, scTransform was found to be particularly effective for datasets produced using the SMART-Seq2 full-length library preparation protocol, while SCnorm was deemed appropriate for smaller datasets. Moreover, significant variations were observed in the computational resource demands of these methods. The outcomes of this study offer valuable guidance for researchers in selecting suitable normalization techniques for single-cell transcriptome data, tailored to the unique attributes of their datasets and research objectives. This selection process is expected to enhance the accuracy and efficiency of scRNA-seq data analysis and facilitate the advancement of single-cell transcriptome research.

## Supporting information

S1 Data Simulation data 1. (ZIP)

S2 Data Simulation data 2. (ZIP)

S3 Data Simulation data 3. (ZIP)

S4 Data Simulation data 4. (ZIP)
